# Yeast-based assays for characterization of the functional effects of single nucleotide polymorphisms in human DNA repair genes

**DOI:** 10.1371/journal.pone.0193823

**Published:** 2018-03-09

**Authors:** Changshin Kim, Jinmo Yang, Su-Hyun Jeong, Hayoung Kim, Geun-hee Park, Hwa Beom Shin, MyungJa Ro, Kyoung-Yeon Kim, YoungJoon Park, Keun Pil Kim, KyuBum Kwack

**Affiliations:** 1 Department of Biomedical Science, College of Life Science, CHA University, Seongnam-si, Gyeonggi-do, Republic of Korea; 2 Department of Life Sciences, College of Natural Sciences, Chung-Ang University, Seoul, Republic of Korea; University of California San Francisco, UNITED STATES

## Abstract

DNA repair mechanisms maintain genomic integrity upon exposure to various types of DNA damage, which cause either single- or double-strand breaks in the DNA. Here, we propose a strategy for the functional study of single nucleotide polymorphisms (SNPs) in the human DNA repair genes *XPD/ERCC2*, *RAD18*, and *KU70/XRCC6* and the checkpoint activation gene *ATR* that are essentially involved in the cell cycle and DNA damage repair. We analyzed the mutational effects of the DNA repair genes under DNA-damaging conditions, including ultraviolet irradiation and treatment with genotoxic reagents, using a *Saccharomyces cerevisiae* system to overcome the limitations of the human cell-based assay. We identified causal variants from selected SNPs in the present analyses. (i) R594C SNP in *RAD3* (human *XPD/ERCC2*) caused severe reductions in the growth rate of mutant cells upon short-wavelength UV irradiation or chemical reagent treatment. (ii) The growth rates of the selected variants in *RAD18*, *YKU70*, and *MEC1* were similar to those of wild-type cells on methyl methanesulfonate and hydroxyurea treated media. (iii) We also assessed the structural impact of the SNPs by analyzing differences in the structural conformation and calculating the root mean square deviation, which is a measure of the discordance of the C_α_ atoms between protein structures. Based on the above results, we propose that these analytical approaches serve as efficient methods for the identification of causal variants of human disease-causing genes and elucidation of yeast-cell based molecular mechanisms.

## Introduction

The maintenance of genomic integrity, mediated by DNA repair pathways, is critical for proper cell cycle progression and cell proliferation. A previous study has reported that up to 10,000 events of DNA damage occur daily in human cells [1]. Accumulation of mutations in DNA sequences can exert detrimental effects on the cell cycle progression and trigger cell death, senescence, or tumorigenesis [2,3]. To prevent the occurrence of such deleterious mutations, cells have acquired various DNA repair mechanisms, most of which are evolutionarily conserved across many species [4]. These DNA repair mechanisms can be categorized into pathways that repair single-strand lesions, double-strand breaks, or interstrand crosslinking.

Repair mechanisms that act on single-strand DNA lesions include the nucleotide excision repair (NER), base excision repair (BER), and mismatch repair (MMR) pathways. NER is a repair system that primarily deals with genetic alterations resulting from helical distortion [5]. The XPC-RAD23B complex in the *xeroderma pigmentosum* complementation group (XP), are involved in NER [6,7]. In BER, enzymes such as glycosylases are involved in the removal of incorrect bases by cleaving the base and restoring the site in a process mediated by AP endonuclease and polymerase β [8]. The MMR pathway repairs the incorrectly matched base pairs. In *Escherichia coli*, the MutS protein detects the misincorporated nucleotide on the daughter strand, based on its methylation status [9]. In addition, MutL activates MutH, which in turn forms a nick at the 5' end of the nearest GATC sequence.

Double-strand breaks (DSBs) are generally repaired via homologous recombination (HR) and non-homologous end joining (NHEJ) [10]. HR appears to be most active in the S and G2 phases, during which the intact sister chromatid acts as the template for successful repair of the damaged strand [11]. In HR-mediated DNA repair, the Mre11, Rad50, and Nbs1 (MRN) complex recognizes the DNA break sites and activates ataxia telangiectasia-mutated (ATM) and ataxia telangiectasia and Rad3-related kinase (ATR) to facilitate the downstream repair process [12,13]. In higher eukaryotes, NHEJ is the dominant mechanism responsible for repairing DSBs in the G1 phase of cell cycle [14]. NHEJ is triggered by the formation of the Ku70/Ku80 heterodimer at DSB ends, wherein it forms a complex with the DNA-dependent protein kinase catalytic subunit (DNA-PKcs), which is mainly present in mammals [15]. Microhomology-mediated end joining (MMEJ) is a recently discovered repair mechanism that differs from other repair pathways in that it utilizes stretches of microhomologous sequences to align broken ends. MMEJ recruits factors such as DNA-PKcs, Ku complex, the MRN complex, or Werner syndrome ATP-dependent helicase, which were previously reported to be involved in various other repair mechanisms [16].

Recent developments have allowed an *in silico* evaluation of the effects of mutations on protein structures. The root mean square deviation (RMSD) is often calculated to estimate the structural similarity between different protein models [17]. RMSD represents the average distance between C_α_ atoms when comparing multiple protein structures. When the structures of two target proteins are positioned in an appropriate orientation, the calculated RMSD value can be converted into the root mean square fluctuation, which is a measure of the difference in conformation of each residue. Thus, RMSD can be used to evaluate the disparity between the two protein structures that are being compared.

In this study, we analyzed the effects of functional single nucleotide polymorphisms (SNPs) in the human DNA repair genes *ATR*, *XPD*/*ERCC2*, *RAD18*, and *KU70*/*XRCC6*, each of which are involved in a different DNA repair pathway. Identification of functional variants in DNA repair genes has drawn research interest since mutations in these genes can lead to higher susceptibility to certain diseases such as Cockayne syndrome, trichothiodystrophy, and various types of cancer [18–21]. In previous studies, screening of variants in candidate DNA-repair genes has been conducted through association studies [20,22]. However, many studies have also reported difficulties in replicating the results of association studies [23–25]. For simplicity, and to facilitate exploratory analysis, we performed a DNA damage-based functional analysis to characterize the SNPs using the budding yeast cell system. The yeast genes selected for this study included *MEC1*, *RAD3*, *RAD18*, and *YKU70*, which are homologs of the human *ATR*, *XPD/ERCC2*, *RAD18*, and *KU70/XRCC6*, respectively. The present study allowed us to examine whether a detailed investigation of SNPs in the genes involved in various DNA repair pathways is useful to evaluate their corresponding impact on cell growth and viability in the presence of DNA-damaging conditions.

## Results and discussion

### *In silico* prediction and selection of SNPs with functional effects

**Fig 1:**
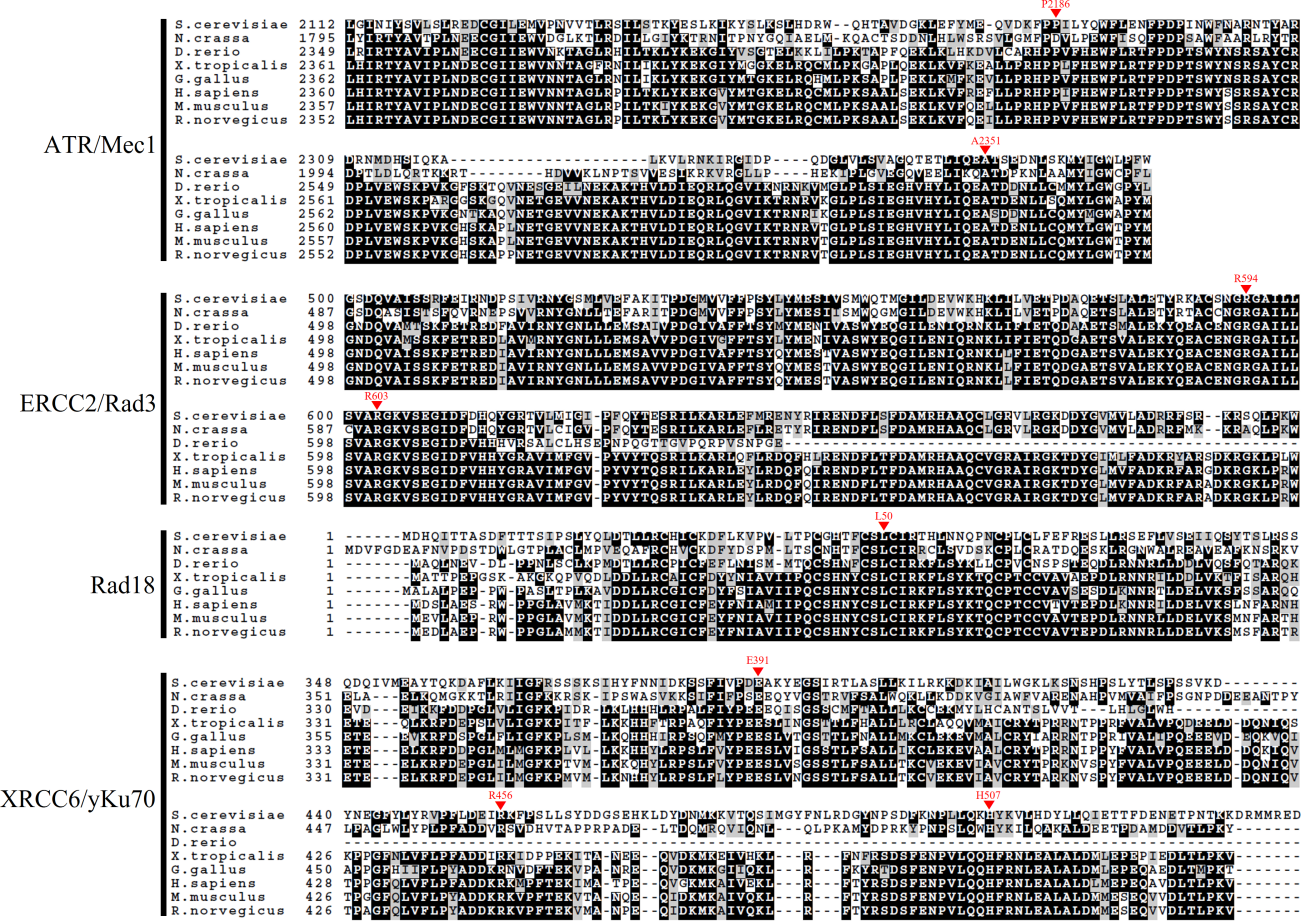
Amino acid sequences alignment of conserved domains from several organisms. SNPs used in this study were conserved among species. Arrow heads indicate SNP regions of human DNA repair genes and the corresponding residue number is included. The amino acid letters in dark shadows represent the sequences of conserved domains.

To identify variants in the human DNA repair genes that can potentially cause diseases such as cancer, we compared the homologous regions of various DNA repair genes between humans and yeast. Homology information of all selected genes was downloaded from the National Center for Biotechnology Information (NCBI, https://www.ncbi.nlm.nih.gov/gene). We aligned the human and yeast sequences using Clustal Omega, using default parameters (http://www.ebi.ac.uk/Tools/msa/clustalo/), and selected the domains and residues that were identical between human and yeast genes [26]. Candidate SNPs were selected based on data obtained from dbSNPs (https://www.ncbi.nlm.nih.gov/SNP/). The functional effects of SNPs in these conserved residues were predicted using the bioinformatics tools Sorting Intolerant From Tolerant (SIFT) (http://sift.jcvi.org/) and Polymorphism Phenotyping-2 (PolyPhen-2) (http://genetics.bwh.harvard.edu/pph2/) [27,28]. We retained variants that were predicted to have significant deleterious effects and shared the same positions in the conserved domains in both humans and *Saccharomyces cerevisiae*. Finally, nine SNPs in four different DNA repair genes, *ATR*, *ERCC2*, *RAD18*, and *XRCC6*, were selected for further analysis (Table 1, Fig 1).

**Table 1:** SNP information.

Gene	Strain	SNP ID	Chromosome: locus	Domain	Ref^a^	Alt^b^	Amino acid change
ATR/MEC1	KBY16	rs33972295	3: 142178118	PIKKc_ATR	G	C	P2186A
	KBY17	rs112027460	3: 142168327	FATC	C	A	A2351S
ERCC2/RAD3	KBY9	rs190678702	19:45353140	HELICc2	G	A	R594C
	KBY10	rs147224585	19:45353139		C	T	R594H
	KBY11	rs140522180	19:45353112		C	T	R603Q
RAD18/RAD18	KBY7	rs189278173	3: 8948561	RING	A	C	L50R
XRCC6/YKU70	KBY3	rs11557356	22:42052946	KU70	G	T	R456M
	KBY4	rs11557352	22:42046881		T	A	E391V
	KBY5	rs4084339	22:42054291		A	T	H507L

^a^ Reference allele.

^b^ Alternative allele.

### Cell growth assay to assess susceptibility to DNA damages

**Fig 2:**
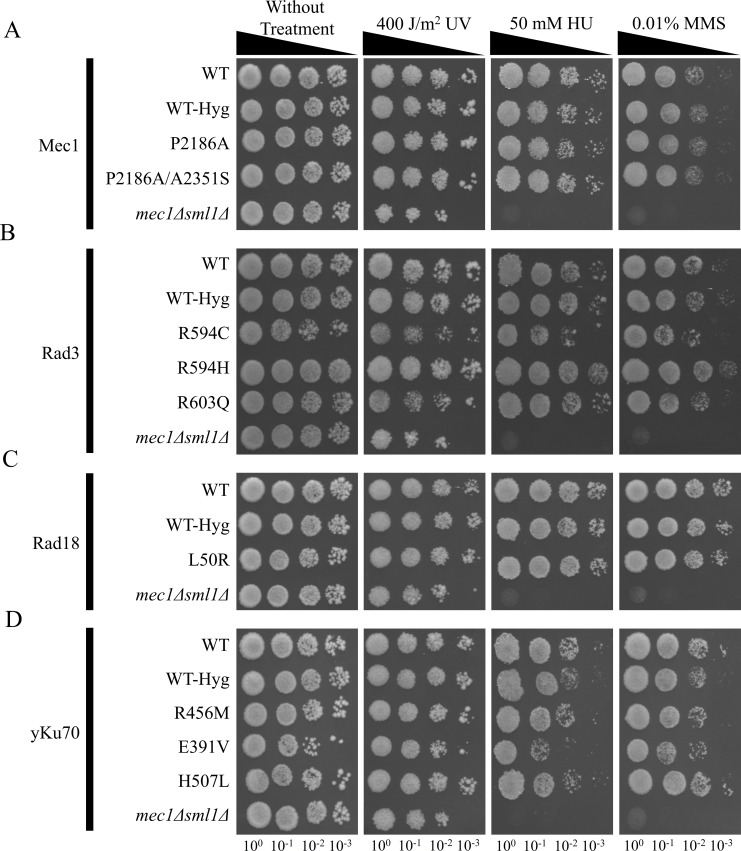
Sensitivity to DNA damaging reagents and UV irradiation of yeast cells carrying gene deletion and mutations. (A-D) Cells were grown overnight, serially diluted tenfold and grown in culture for 48 h and analyzed after UV irradiation or treatment with chemical reagents. (A) Mec1, Mec1 P2186A, Mec1 P2186A/A2351S, and *mec1Δ sml1Δ* cells. (B) Rad3, Rad3 R594C, Rad3 R594H, Rad3 R603Q, and *mec1Δ sml1Δ* cells. (C) Rad18, Rad18 L50R, and *mec1Δ sml1Δ* cells. (D) yKu70, yKu70 R456M, yKu70 E391V, yKu70 H507L, and *mec1Δ sml1Δ* cells. The R594C mutant showed lower growth rates in all conditions when compared to WT and WT-Hyg at low cell concentrations. The E391V mutants were visible as fewer and smaller colonies in all treated conditions when compared to other mutant strains and WT strain.

**Fig 3:**
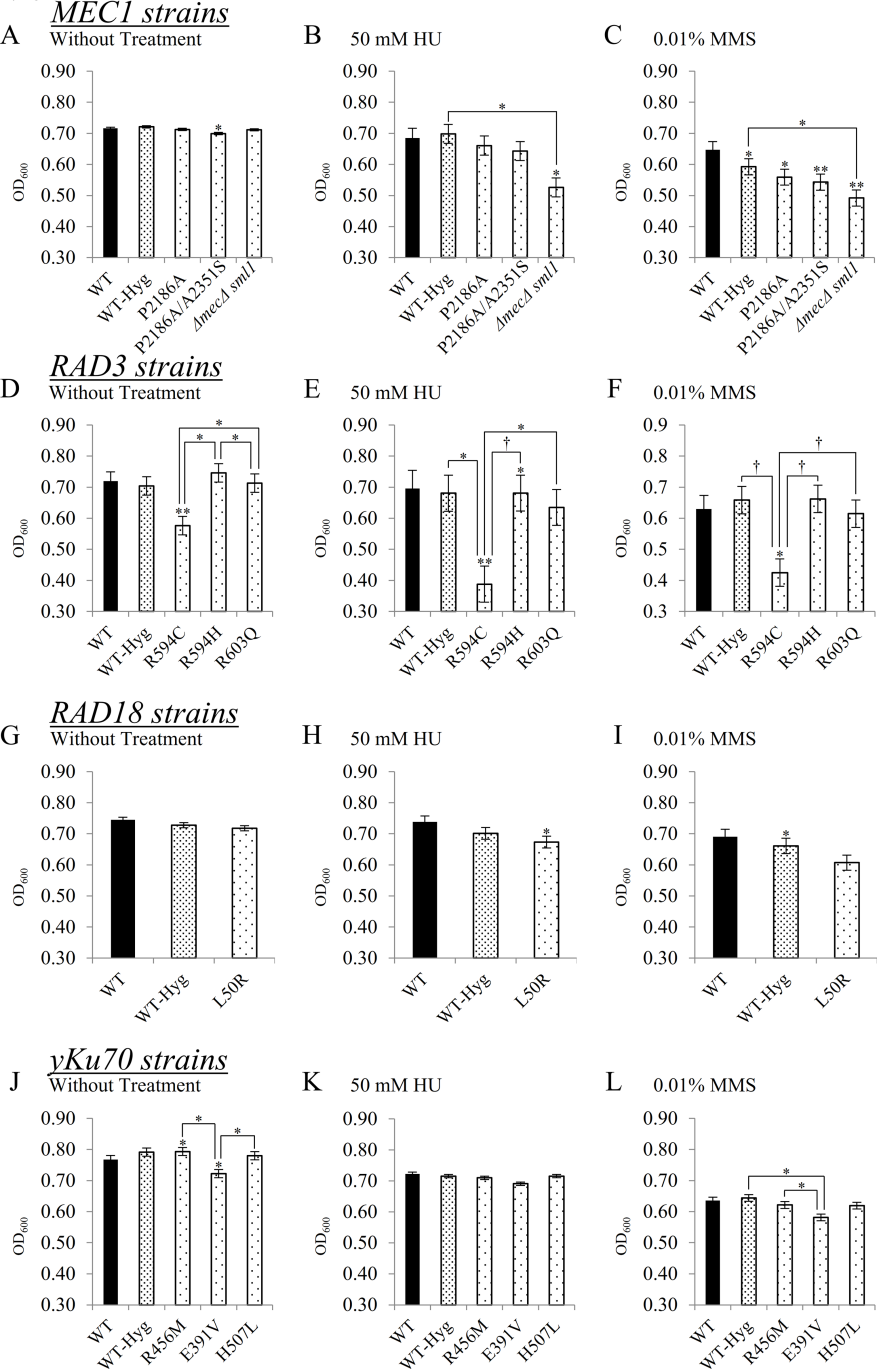
Cell growth of *MEC1*, *RAD3*, *RAD18*, *yKu70*, and mutant cells after HU or MMS treatment. (A) Under conditions with no genotoxic treatment, the *MEC1* P2186A/A2351S double mutant showed a lower growth rate compared to WT. (B) Only *mec1Δ sml1Δ* showed significantly slower growth under HU treatment. (C) Under MMS treatment, all strains showed slower growth compared to WT. *mec1Δ sml1Δ* showed significantly reduced growth compared to WT-Hyg in both HU and MMS treatment conditions. (D) Under conditions without treatment, significant differences in growth were observed between the *RAD3* WT and mutant strains. Only the *RAD3* R594C mutant showed significantly reduced growth compared to WT. (E) Under 50 mM HU treatment, *RAD3* R594C cells showed substantially reduced growth compared to WT, WT-Hyg, and other mutant strains. (F) In 0.01% MMS-treated liquid YPD, the *RAD3* R594C mutant showed substantially reduced growth compared to WT, WT-Hyg, and other mutant strains. (G) Under conditions without treatment, no significant differences in growth were observable in WT and mutant strains. (H) Under HU treatment, *RAD18* L50R did not show significantly reduced growth compared to WT-Hyg. (I) Under MMS treatment, the *RAD18* L50R mutant strain did not show any significant difference in growth when compared to WT and WT-Hyg. (J) In YPD plates without genotoxic agents, *yKu70* R456M showed higher growth rate compared to WT. *yKu70* E391V displayed considerably reduced growth compared to WT and other mutant strains. (K) No significant growth differences were observed in WT and mutant strains when cultured in YPD containing HU. (L) Under MMS treatment, *yKu70* E391V showed reduced growth compared to the WT-Hyg and *yKu70* R456M strains. Cells were grown for 8 h in varying conditions, after which OD_600_ values were measured. Student’s paired *t*-test was performed on WT, WT-Hyg, and all mutant strains. WT: wild-type; WT-Hyg: wild-type with hygromycin vector. *: P-value < 0.05. **: P-value <0.01. * and ** without comparative bars indicate statistically significant differences compared to WT. X axis indicates mutant strain and Y axis indicates OD_600_ value.

To examine the growth characteristics of yeast cells harboring mutations in the *MEC1* gene, mutant strains were cultured on yeast extract-peptone-dextrose (YPD) plates, treated with various DNA damaging agents, and subsequently subjected to growth assays (Fig 2A). Under ultraviolet (UV) irradiation, the *mec1Δ sml1Δ* strain showed less growth at all cell concentrations when compared to the other mutants and the wild-type with hygromycin marker (WT-Hyg) strain (Fig 2A) [29,30]. After UV irradiation, the cell growth of *mec1Δ sml1Δ* was similar to that of WT at 10^−2^ concentrations, which suggests an about 10-fold reduction in cell viability due to UV irradiation. Further, among the Mec1 P2186A/A2351S double mutant strain and Mec1 P2186A strain, there was no significant difference in the colony number on 50 mM hydroxyurea (HU) treated media (Fig 2A). The most defective cell growth was observed when cells were treated with 0.01% methyl methanesulfonate (MMS), in which no clear colonies were visible in all strains at the concentration of 10^−3^ cells. Moreover, the *mec1Δ sml1Δ* strain did not form any visible colonies at all cell concentrations in plates treated with either HU or MMS (Fig 2). Since mutant phenotypes cannot be distinguished from WT in this assay, we carried out a detailed cell growth analysis, for which the cells were cultured in liquid YPD media and OD_600_ values were measured after 8 h of incubation (Fig 3). In untreated YPD plates, no significant changes were observable between WT and mutant strains (Fig 3A). In 50 mM HU or 0.01% MMS treated conditions, the growth of both P2186A and P2186A/A2351S cells did not show any significant change when compared to both WT strains (Fig 3B and 3C). Although this result is not consistent with the colony forming assay described in Fig 2A, this culture-based assay might have provided more accurate information. Furthermore, under HU and MMS treatment conditions, growth rates of *mec1Δ sml1Δ* cells also showed significantly reduced growth compared to the WT strain (Fig 3A–3C).

The *RAD3* R594C mutant strain formed relatively fewer colonies at all cell concentrations and culture conditions when compared to other strains (Fig 2). *RAD3* R594H and R603Q cells formed a consistent number of colonies regardless of cell concentration. Upon exposure to UV irradiation, R594C mutants formed fewer colonies compared to other strains, whereas the R594H mutants did not show any significant difference in colony size and number compared to those of the WT strains (Fig 2B). Upon treatment with 50 mM HU, R594C mutants exhibited slower growth rates compared to other strains, and almost no growth was observed at the 10^−3^ cell concentration. In contrast, the growth rates of R603Q cells were similar to those of the WT cells. The same results were observed when cells were treated with 0.01% MMS, wherein the R594H mutants formed the densest colonies at the 10^−2^ concentration compared to other cells. However, we also observed that R594H mutation did not affect Rad3’s DNA repair activity (Fig 3D–3F). In addition, the R603Q mutants did not show significant differences in colony characteristics compared to the WT strain, whereas R594C mutants formed the smallest colonies (Fig 2B). A comparison of all strains under conditions without treatment revealed that R594C mutants had significantly lower OD_600_ values compared to those of the WT strain (Fig 3D–3F). Moreover, upon treatment with 50 mM HU or 0.01% MMS, R594C mutants showed significantly slower growth rates when compared to the WT cells (Fig 3D–3F). In particular, in conditions with 0.01% MMS treatment, the R594C cells showed the largest differences in growth rates compared to WT cells (Fig 3D–3F). In humans, mutations in the R594 residue have often been correlated with xeroderma pigmentosum, which is generally characterized by an increased susceptibility to UV radiation [31]. Thus, the R594C variant possibly results in a defective Rad3-mediated DNA repair pathway.

The results of the growth rate assay revealed that the *RAD18* L50R mutant showed no significant differences in growth rates under conditions without genotoxic treatment at 10^−2^ cell concentrations (Fig 2C). Upon treatment with HU or MMS, the *RAD18* L50R mutants also showed no significant differences when compared to WT-Hyg (Fig 3G–3I). Furthermore, in the liquid culture-based growth analysis, the L50R mutant strain exhibited no significant growth defects when compared to WT strain, implying that the presence of the L50R SNP does not result in functionally drastic damaging effects as predicted in the colony forming assay (Fig 3G–3I).

The assays for *yKu70* mutants showed that the E391V mutant showed significantly slower growth in conditions treated without any damaging agents (Fig 3J–3L). Thus, E391V might be toxic for cellular progression independent of the non-homologous end joining pathway. Notably, in media containing 50 mM HU, the E391V strain exhibited a reduction of growth rate, but not statistically significant, when compared to the WT and WT-Hyg strains (Fig 3J–3L). However, we could not exclude the possibility that the yKu70 E391V mutation interrupts the Ku pathway, which also requires further analysis for a more precise conclusion.

### *In silico* analysis of the structural impact of mutations

**Fig 4:**
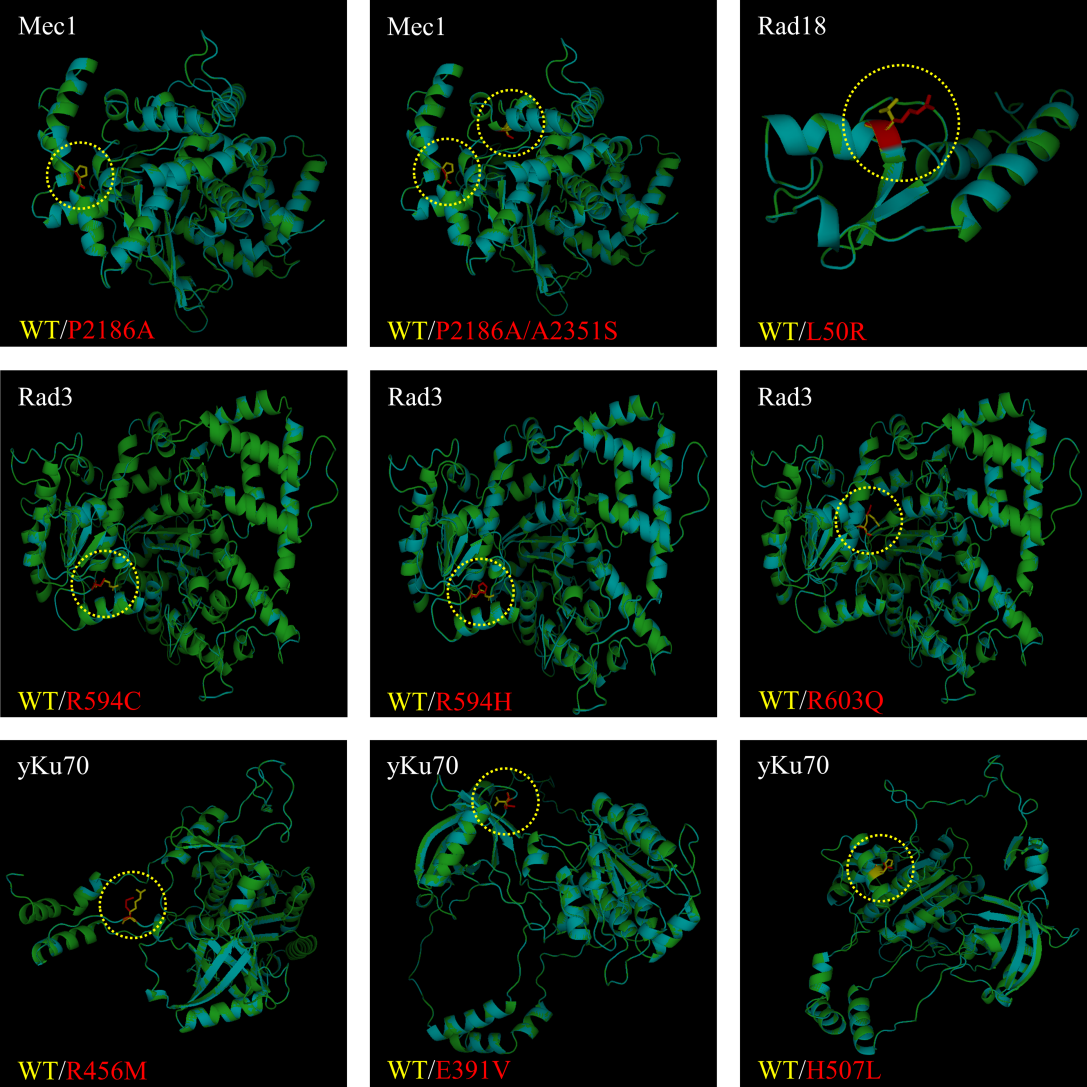
Protein 3D structural modeling of Mec1, Rad3, yKu70, and Rad18. Wild-type and mutant proteins were superimposed for comparison. P2186A and P2186A/A2351S are *MEC1* mutants. L50R is a *RAD18* mutant. R594C, R594H, and R603Q are *RAD3* mutants. R456M, E391V, and H507L are *yKu70* mutants. Green: wild-type protein structure. Cyan: mutant protein structure. Yellow: wild-type amino acid at the point mutation site. Red: mutant strain amino acid at the point mutation site.

The human *ATR* gene is homologous to the yeast *MEC1* gene and is a member of the phosphatidylinositol 3-kinase (PI3K)-related kinase (PIKK) family of proteins, which contain a FAT/kinase domain/FATC structure that is essential for kinase activity [32]. Mec1 can physically interact with Rad53. Also, Rad53 is activated by the Mec1 kinase-dependent phosphorylation [33]. To evaluate the functional effects of the target SNPs at the protein level, structures of the proteins from each of the WT and mutant strains were modeled (Fig 4). Structural modeling was limited to the PI3K/PI4K and FATC domains due to the lack of accurate whole-protein X-ray crystallography data. The results showed that mutations in the FATC domain did not significantly alter protein conformation (Fig 4).

**Fig 5:**
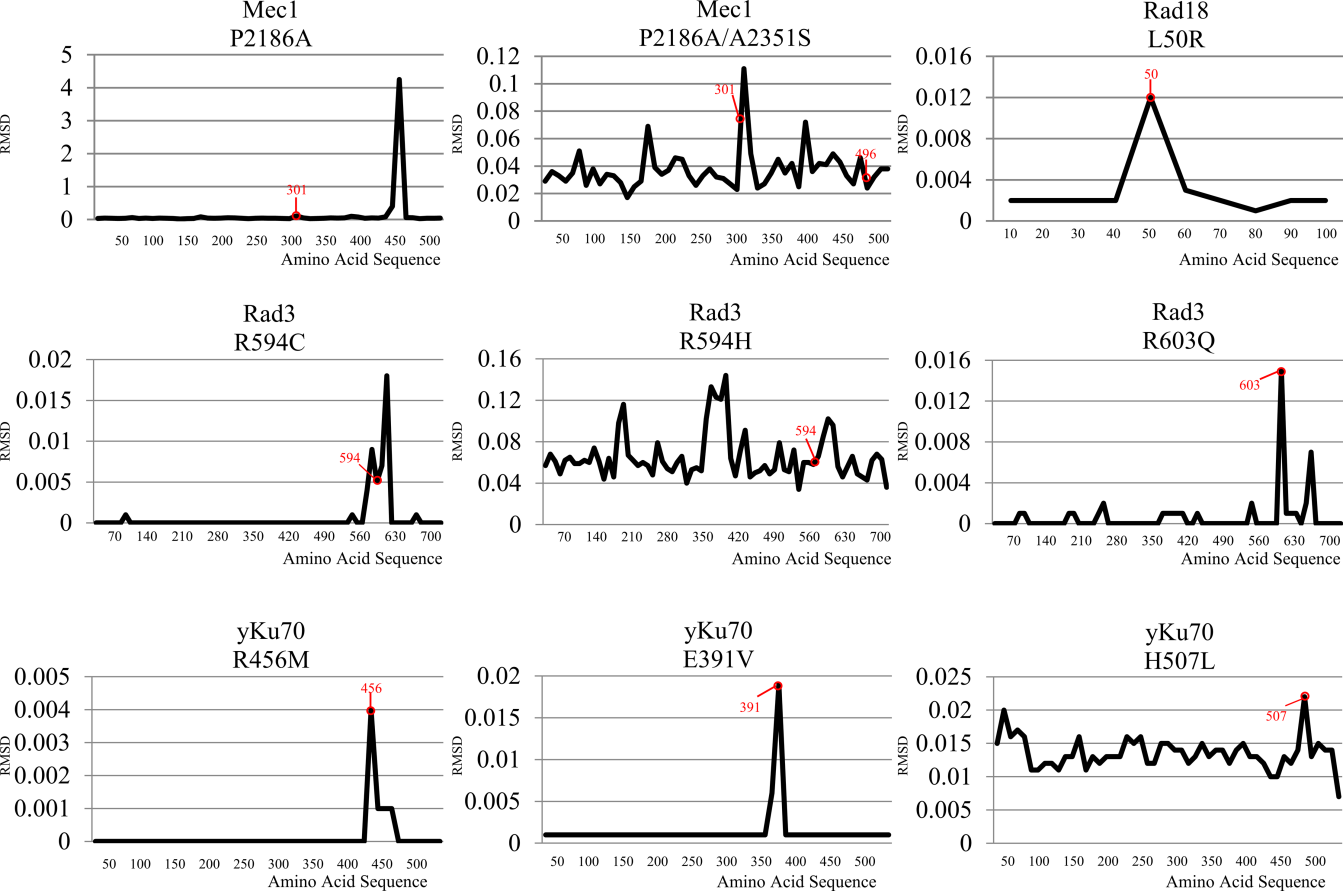
RMSD calculations for all residues of Mec1, Rad3, yKu70, and Rad18. Comparison between the amino acid sequences of Mec1, Rad3, yKu70, and Rad18 was conducted between the wild-type and mutant strains. The vertical axis represents the calculated RMSD value. The horizontal axis represents domain residue number. Considering that not all protein structures were complete, RMSD was calculated in protein domains whose structures have been fully determined, which led to differences in the numbers of displayed domain residues and actual SNP residues in some proteins. The residues of mutations are marked in red circles.

RMSD is a measure of the disparity between the target protein sequence and reference protein sequence. For comparison of RMSD, each target sequence containing the target point mutation was compared to the corresponding reference sequence from *S*. *cerevisiae*. The calculated RMSD values between the WT and mutant Mec1 proteins revealed significant torsion at 30 residues downstream of the Mec1 P2186A mutation site (Fig 5). As previously described by Reiersen, the characteristic change in torsion angle induced by proline influences the overall stability of structural motifs [34]. The proline-to-alanine substitution might have caused the increase in structural torsion at the downstream residues, because of larger gaps formed at more distant residues than directly at the site of angular change. Interestingly, the double mutant protein showed normalization of the previous peak, to the level equivalent to that of the average RMSD, whereas comparison of RMSD values between P2186A and P2186A/A2351S did not result in peak reduction (Fig 5). Thus, the differing results could have occurred due to limited information from the structure of Mec1, since only domains at the C-terminus were analyzed.

Rad3 R594C and R603Q showed slightly higher RMSD values at the mutation site (Fig 5). In contrast to the insignificant difference observed between R594H and WT in the growth assays, the R594H mutant showed a dramatic overall change in the structure of the whole protein when compared to that of the WT (Fig 4). The RMSD between Rad3 R594C and R594H showed an almost tenfold difference; thus, a SNP at the same position could exert a huge impact on the overall protein conformation (Fig 5). The large difference in the RMSD between R594C and R594H could be explained by the characteristics of the original and mutated amino acids. In R594H, both arginine and histidine are basic amino acids that exhibit similar properties and do not appear to interact with other residues despite the bulky ring of histidine. On the other hand, the mutation from arginine to cysteine is expected to exert a more significant change in protein structure because of the difference in the properties of the functional groups (Fig 5). Based on the RMSD results, we hypothesized that the observed differences between R594C and R594H during DNA repair were caused by changes in the binding affinity of Rad3 with DNA, as a result of conformational changes. Further studies on the mutant proteins could investigate whether the mutations caused changes in protein-DNA binding affinities. Furthermore, as previously reported by Barwell *et al*. and Maines *et al*., Rad3 is known to bind to Ssl1 and Ssl2 [35,36]. Mutations in Rad3 could have altered this interaction, which in turn resulted in the observed growth rate difference when compared to the WT strain.

The Rad18 L50R mutant had a higher RMSD value at the mutated residue, although the calculation was limited to the N-terminal region, which represents a small part of the whole protein (Fig 5). Further determination of the protein structure will allow more accurate RMSD calculations. The RMSD values of yKu70 E391V and R456M were found to be slightly higher near the point mutation, whereas that of H507L showed inconsistent values in sites with the same residues (Fig 5).

As described by Nigham and Hsu, detection of conformational changes via RMSD has certain limitations, such as the tendency to produce noisy data [37]. The low overall RMSD of the protein also contributes to the difficulty in distinguishing the actual structural changes from background noise. A previous study has reported the difficulties in stabilizing RMSD data based on the largest common point set problem and the minimum aligned distance problem [38]. Moreover, the range RMSD query problem occurs during the calculation of the difference between RMSDs of two aligned substructures with no gaps, which is essential to obtain more reliable estimates of the discrepancies between protein structures [39]. Although the calculation of RMSD between proteins provides substantial information on protein conformation, predicted protein structures provide limited accuracy. Further studies need to be carried out for the experimental determination of protein structure through conventional methods, such as nuclear magnetic resonance or X-ray crystallography. These standard methods can be used to determine actual RMSD values and derive deeper insights on the effects of structural changes on protein function by investigating the interaction between previously known domains and motifs (Fig 1). The development of new structure prediction algorithms with higher coverage would also facilitate more accurate RMSD calculations and enable more detailed investigation of protein mutations and their corresponding structural effects.

## Materials and methods

### Yeast strains and culture media

Yeast strains generated in this study were derived from the SK1 strain (Table 2). All mutants were generated using a site-directed mutagenesis method. Cells were grown on YPD (1% yeast extract, 2% bacto-peptone, and 2% dextrose) (Becton Dickinson (BD), Franklin Lakes, NJ, USA). YPD plates were treated with reagents, 50 mM hydroxyurea (Sigma-Aldrich, St. Louis, MO, USA) and 0.01% methyl methanesulfonate (Sigma-Aldrich). After seeding yeast on YPD, UV irradiation was performed using a UV crosslinker CL-1000 (UVP, Upland, CA, USA) system. For transformation, *E*. *coli* strain DH-5 alpha (Enzynomics, Daejeon, South Korea) cells were grown on LB plates containing tryptone (AMRESCO, Solon, OH, USA), yeast extract, agar (BD), NaCl (Sigma-Aldrich), and ampicillin (AMRESCO).

**Table 2:** Yeast strains used in this study.

Strain	Genotype	Source
KBY1	*ho*::*hisG*, *leu2*::*hisG*, *ura3 (sam-pstI)*, *KU70*::*hygB*	this study
KBY3	*ho*::*hisG*, *leu2*::*hisG*, *ura3 (sam-pstI)*, *ku70-R456M*::*hygB*	this study
KBY4	*ho*::*hisG*, *leu2*::*hisG*, *ura3 (sam-pstI)*, *ku70-E391V*::*hygB*	this study
KBY5	*ho*::*hisG*, *leu2*::*hisG*, *ura3 (sam-pstI)*, *ku70-H507L*::*hygB*	this study
KBY6	*ho*::*hisG*, *leu2*::*hisG*, *ura3 (sam-pstI)*, *RAD18*::*hygB*	this study
KBY7	*ho*::*hisG*, *leu2*::*hisG*, *ura3 (sam-pstI)*, *rad18-L50R*::*hygB*	this study
KBY8	*ho*::*hisG*, *leu2*::*hisG*, *ura3 (sam-pstI)*, *RAD3*::*hygB*	this study
KBY9	*ho*::*hisG*, *leu2*::*hisG*, *ura3 (sam-pstI)*, *rad3-R594C*::*hygB*	this study
KBY10	*ho*::*hisG*, *leu2*::*hisG*, *ura3 (sam-pstI)*, *rad3-R594H*::*hygB*	this study
KBY11	*ho*::*hisG*, *leu2*::*hisG*, *ura3 (sam-pstI)*, *rad3-R603Q*::*hygB*	this study
KBY15	*ho*::*hisG*, *leu2*::*hisG*, *ura3 (sam-pstI)*, *MEC1-HygB*	this study
KBY16	*ho*::*hisG*, *leu2*::*hisG*, *ura3 (sam-pstI)*, *mec1-P2186A*::*HygB*	this study
KBY17	*ho*::*hisG*, *leu2*::*hisG*, *ura3 (sam-pstI)*, *mec1-P2186A/A2351S*::*HygB*	this study
KKY134	*ho*::*hisG*, *leu2*::*hisG*, *ura3 (sam-pstI)*, *sml1 Δ*::*hphMX4*, *mec1 Δ*::*leu2*	this study
KKY153	*ho*::*hisG*, *leu2*::*hisG*, *ura3 (smaI-pstI)*	this study

### Construction of plasmids

To extract genomic DNA, yeast cells were treated with cell lysis buffer (2% Triton X-100, 1% SDS, 100 mM NaCl, 20 mM Tris, pH 7.6, and 1 mM EDTA, pH 8.0), disrupted using glass beads, and treated with RNase A (Sigma-Aldrich). Site-directed mutagenesis was then performed via inverse polymerase chain reaction (PCR) method using the extracted genomic DNA as a template. Primers used for generating the mutants contained the point mutation and restriction enzyme site (Table 3). PCR products were inserted into the pFA6a-hphNT1 plasmid using the restriction enzymes *Hind*III and *PacI* (New England Biolabs, Ipswich, MA, USA). After transformation, *E*. *coli* cells were cultured and selected on LB media containing ampicillin. The resulting PCR products were transformed into yeast and selected on YPD plates containing hygromycin. All the primers were manufactured by Integrated DNA Technologies (IDT) (Coralville, IA, USA). Each control wild-type strain (WT-Hyg) was constructed by amplifying the hygromycin gene from pFA6a-hphNT1 (S1 Fig).

**Table 3:** Primer sequences used for PCR.

Gene	Primer Name	Sequence
MEC1	Ins MEC1 F^a^^,^^b^	ATATAAGCTTGCAATCTTGTACCAATGGTTTTTAGAAAAC
	Ins MEC1 R^c^	ATATTTAATTAATTACCAAAATGGAAGCCAACCAATATACAT
	HR WT F^d^	TAAATTTCCTGCAATCTTGTACCAATGGAATTTAG
	HR WT R	CCTGCAGTGATGGTTAGATCAAGAGGAAGTTCGTCTGTTGCCGAAAATGGTGGAAAGTCGCAAAACCTTCTCAAGCAAGGTTTT
	HR P2186A F	TTTCCTGCAATCTTGTACCAATGGTTTTTAGTCGAGTTTTACATGGAACAGGTAGATAAA
	HR A2351S F	GCTGGCCAAACAGAAACATTGATCCAAGAATCAA
	WT Single R^e^	GATCAATGTTTCTGTTTGGCC
	P2186A Single R	
	A2341S Single R	
	P2186A Double R^f^	TTGTCTTCTGATGTTGATTCTTG
	A2351S Double R	
	Seq MEC1 F^g^	GGAAATGGTACCGAATGTTGTAACTTTAAGATCTATTCTTTCTACAAAGTACGAA
	Seq MEC1 R	ACGAGAAGTCAATCTTTCCAATAACTTGTCTAAAACCACCATAATAATTTTC
RAD3	Ins RAD3 F	ATATAAGCTTGGGTGTGGGGCAATTTTGCTT
	Ins RAD3 R	ATATTTAATTAATCACTGCATTTCTATGTCTTCATCTTCATC
	HR WT F	CTCAAATGGGCGTGGGGCAATTTTGCT
	HR R594C F	CCTTAGAAACCTATAGAAAGGCTTGCTCAAATGGGTGTGGGGCAATTTTGCTTTC
	HR R594H F	CCTTAGAAACCTATAGAAAGGCTTGCTCAAATGGGCATGGGGCAATTTTGCTTTC
	HR R603Q F	GGGCGTGGGGCAATTTTGCTTTCTGTTGCTCAAGGAAAGGTATCTG
	HR WT R	CTTGCGGCTATTTAATCTAATTGTGATATATACAGTTTATAGCAAAAGCGTATCATTGCACAAAACCTTCTCAAGCAAGGTTTTCAG
	Seq RAD3 F	AAAGTATTGTTTCAATGTGGCAAACAATGGGTA
	Seq RAD3 R	GATTCATATCTTGACGCAGATAAAGAGTTGTCCTTTG
RAD18	Ins RAD18 F	ATATAAGCTTGTTCCCGTTGTATTAGAACACATTTGAA
	Ins RAD18 R	ATATTTAATTAATTAATTGTTACCGGGTGGGTCTTTAC
	HR WT F	CATTTTGTTCCCTTTGTATTAGAA
	HR L50R F	AAAGTCCCCGTCTTAACACCTTGTGGCCATACATTTTGTTCCCGTTGTATTAGAACACATTTGAATAACCA
	HR WT R	AAATTATTAATTAACAAATGTGCACAAGCTAACAAACAGGCCTGATTACATATACACACCCAAAACCTTCTCAAGCAAGGTTTTCAG
	Seq RAD18 F	TTGGATACACTTTTGAGATGTCACA
	Seq RAD18 R	TAACGAAATAATATATATTAATGTTAAATATGATTACATA
YKU70	Ins YKU70 F	ATATAAGCTT TTCTCACCCTCAAGCGTGAAGG
	Ins YKU70 R	ATATTTAATTAA TTATATATTGAATTTCGGCTTTTTATCAAAGG
	HR WT F	GCTTAAATCAAATTCACATCCTTCACTATATACGTTATCACCCTCAAGCGTGAAGG
	HR WT R	CAAATACCCTACCCTACCTACATATTTTATGTAACGTTATAGATATGAAGGATTTCATTCGTCTCAAAACCTTCTCAAGCAAGGTTTTCAG
	HR R456M F	GGACTACAACGAAGGATTTTATCTCTACAGGGTTCCATTCCTAGACGAAATTATGAAATTTCC
	HR E391V F	GGGATACTTTAACTTGAGGGATGGATATAACCCATCCGATTTCAAAAACCCACTATTACAAAAACTTTACAAAGTTTTAC
	HR H507L F	CGTTCATCGAGCAAATCGATACACTATTTCAATAACATAGACAAAAGTTCGTTTATCGTACCCGATGTAGCAAAATATGAAGG
	Seq YKU70 F	CCTTGGCTTCTTTATTAAAAATTTTGAG
	Seq YKU70 R	GATTACTGTCGTGCATAAATATCTTGC

^a^ Ins: primers used for vector insertion and include restriction enzyme site.

^b^ F: forward primer.

^c^ R: reverse primer.

^d^ HR: primers used for homologous recombination during transformation from yeast to either vector or *E*. *coli*.

^e^ Single: primer used to generate WT, P2186A, and A2351S single mutant strains.

^f^ Double: primer used to generate P2186A/A2351S double mutant strain.

^g^ Seq: primers used for sequencing.

### Analysis of UV sensitivity

Cells were pre-cultured in YPD medium overnight, after which 500 μL of each strain was added to 4 mL of fresh YPD medium and incubated for 3 h. Each sample was adjusted to an OD600 value of 0.1. Cells were serially diluted tenfold with distilled water, spotted onto YPD plates, and irradiated with UV at 400 J/m^2^ [40]. Plates were observed every 12 h until 48 h and photographed.

### Analysis of sensitivity to DNA damaging agents

Cells were pre-cultured in YPD medium overnight, after which 500 μL of each strain was added to 4 mL of fresh YPD medium and incubated for 3 h. Each sample was adjusted to an OD_600_ value of 0.1, serially diluted tenfold with distilled water, and spotted onto YPD plates pretreated with 50 mM HU [41]or 0.01% MMS [42]. Plates were incubated and observed every 12 h until 48 h and photographed. To compare the growth rates, each strain was cultured overnight in YPD, after which 800 μL of sample was added to 4 mL of fresh YPD medium containing 50 mM HU or 0.01% MMS. Cells were cultured in a medium for 12 h and OD_600_ was measured every 2 h for assessing growth rate.

### Statistical analysis

OD_600_ values were measured at 8 h after incubation. Student’s paired *t*-test was conducted to determine statistically significant differences between mutant and control strains. All statistical tests were performed using R (version 3.2.2, https://cran.r-project.org/). All experiments in this study were performed using *MEC1*, *RAD3*, *RAD18*, and *YKU70* mutant strains and were independently repeated thrice. The *MEC1* deletion strain *mec1Δsml1Δ* was used as a negative control.

### Calculation of the impact of target SNPs on protein structure using bioinformatics tools

To assess the impact of SNPs on the proteins, we compared the protein structures using PyMol (The PyMOL Molecular Graphics System, Version 0.99, Schrödinger, LLC). After structural modeling, proteins harboring the target SNPs were rendered and superposed with WT proteins. Sites that varied between the models were marked yellow for WT and red for mutant, respectively. Additionally, the RMSD value of each mutant protein was calculated to evaluate the impact of the SNP-induced conformational changes in each protein.

## Supporting information

S1 Fig Schematic diagram of generating mutant constructs of MEC1 and introducing into S. cerevisiae. The MEC1 gene of yeast was amplified via PCR using primers (1) and (2) marked as purple arrows, and cut using restriction enzymes PacI and HindIII. Products were inserted into the PFA6a-hphNT1 vector and ligated. The PCR product and selective marker were transformed back into the yeast genome via homologous recombination using primers (3), (4), (5) and (6) marked as blue arrows. After homologous recombination, the results were sequenced using sequencing primers (7) and (8) marked as green arrows. All primers are labeled as in Table 3. (1) Ins MEC1 F; (2) Ins MEC1 R; (3) HR WT F; (4) HR P2186A F; (5) HR A2351S F; (6) HR WT R; (7) Seq MEC1 F; (8) Seq MEC1 R.
(TIF)
