# Supplementary figures and images for: Yeast-based assays for characterization of the functional effects of single nucleotide polymorphisms in human DNA repair genes
Source: PLoS One. 2018 Mar 9;13(3):e0193823. doi: 10.1371/journal.pone.0193823 (PMC5844570; doi:10.1371/journal.pone.0193823)

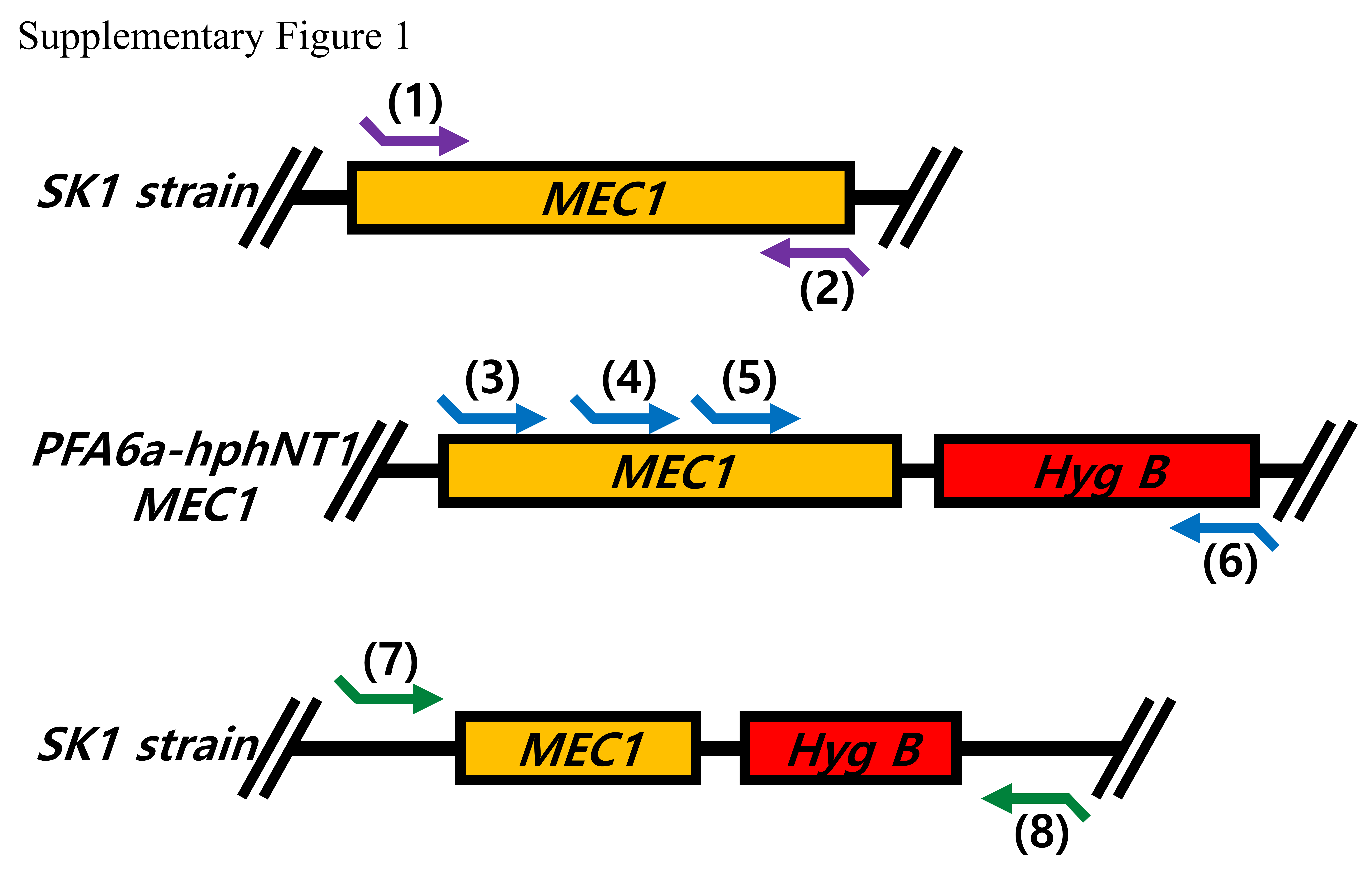

Supplement: S1 Fig — The MEC1 gene of yeast was amplified via PCR using primers (1) and (2) marked as purple arrows, and cut using restriction enzymes PacI and HindIII. Products were inserted into the PFA6a-hphNT1 vector and ligated. The PCR product and selective marker were transformed back into the yeast genome via homologous recombination using primers (3), (4), (5) and (6) marked as blue arrows. After homologous recombination, the results were sequenced using sequencing primers (7) and (8) marked as green arrows. All primers are labeled as in Table 3. (1) Ins MEC1 F; (2) Ins MEC1 R; (3) HR WT F; (4) HR P2186A F; (5) HR A2351S F; (6) HR WT R; (7) Seq MEC1 F; (8) Seq MEC1 R. (TIF) [file pone.0193823.s001.tif]
